# A Diffusion-Based pH Regulator in Laminar Flows with Smartphone-Based Colorimetric Analysis

**DOI:** 10.3390/mi9120616

**Published:** 2018-11-23

**Authors:** Wei Wang, Zhi Zeng, Wei Xu, Wenming Wu, Wenfeng Liang, Jia Zhou

**Affiliations:** 1 ASIC and System State Key Laboratory, School of Microelectronics, Fudan University, Shanghai 200433, China; 14110720019@fudan.edu.cn (W.W.); 10300720149@fudan.edu.cn (Z.Z.); wei_xu@fudan.edu.cn (W.X.); 2 State Key Laboratory of Applied Optics, Changchun Institute of Optics, Fine Mechanics and Physics (CIOMP), Chinese Academy of Sciences, Changchun 130033, China; 3 School of Mechanical Engineering, Shenyang Jianzhu University, Shenyang 110168, China; liangwf@sjzu.edu.cn

**Keywords:** pH regulator, diffusion, laminar flows, smartphone, colorimetric analysis

## Abstract

A strategy for an on-chip pH regulator is demonstrated computationally and experimentally, based on the diffusion characteristics of aqueous ionic solutions. Micro-flows with specific pH values are formed based on the diffusion behaviors of hydrogen and hydroxide ions in laminar flows. The final achieved pH value and its gradient in the channel can be regulated by the amount of ions transported between laminar flows, and the experimental results can be further generalized based on the normalized Nernst-Planck equation. A smartphone was applied as an image capture and analysis instrument to quantify pH values of liquids in a colorimetric detection process, with monotonic response range of ~1–13.

## 1. Introduction

The significant effects of hydrogen ions on the activities or functions of bio-samples (such as proteolysis [1,2], culture of bio-samples [1] and so on) make it necessary to form an environment of specific hydrogen ionic concentration. Take currently available polymerase chain reaction (PCR) devices as an example: pH variation can in principle denature/renature DNA duplexes to trigger chain reactions in a similar way to temperature cycling [3,4]. This alternative approach has not been fully explored, which is possibly due to the fact that changing the solution pH in an automated way is difficult for most microfluidic designs.

When transferring conventional macroscale techniques to microfluidic systems, valves or membranes are usually needed to control the liquid flow or electro-dialysis process, wherein a substantial time is necessary for solutions to get well mixed [5,6,7]. The water electrolysis technique has received attention for its easy manipulation and good compatibility with microfluidic systems [2,8,9,10]. However, gas bubbles (H_2_ and O_2_) formed in the electro-chemical process could introduce turbulence into the microfluidics [1]. Moreover, direct contact between electrodes and fluidic samples increases the possibility of cross-contamination. A technique using bipolar membranes [1,11] is regarded a superior method. However, a voltage bias should be consistently applied and the throughput is restricted by its feature time [2,9,12]. It could be difficult to generate a continuous spatial gradient of pH for applications like isoelectric focusing [12,13] while maintaining a specific and stable pH in a confined environment at the same time [10]. Fortunately, a laminar system offers useful features to overcome the difficulties mentioned above. The lateral migration of commonly used bio-samples could be suppressed by the drag force [14,15], while ionic electrolytes like hydrogen and hydroxide ions could diffuse through the concentration gradient and redistribute within the channel [16,17,18]. It provides a feasible mechanism to regulate the aqueous concentration of hydrogen and hydroxide ions, while avoiding the unnecessary migration of bio-samples. However, the ionic redistribution process has not yet been demonstrated in detail for microscale pH regulators.

To make the whole study more practical, it would be better if one could monitor the pH value of the output liquid. The results could be utilized to guide the tuning of operating parameters. For example, increase the amount of alkaline material introduced when the detected pH value was lower than the desired one, and vice versa. For the detection, paper-based microfluidic devices have emerged in recent years [19,20,21,22]. One reason for their popularity is that they can provide simple, cheap, and fast ways for chemical or biological screenings in resource-limited environments [23,24]. However, these original versions are normally viewed by the naked eye, which makes it difficult to achieve precise results [25]. To improve accuracy, bulky apparatuses like a video camera [26], digital color analyzer [27], scanner [28], and custom portable reader [21] have been introduced. But they still need specialized instrumentation in one way or another, and all execute image analysis with a computer. Thanks to the rapid growth of telecommunication infrastructure, a smartphone could act as a promising tool for lab-on-a-chip diagnostics in processes such as reading, recording, analyzing, and transferring data [29,30]. Microfluidic systems based on mobile technology have gained much attention recently for point-of-care analysis of sodium [31], vitamin D [32], salmonella [33], cholesterol [34,35], hydrogen peroxide [36]. and so on. Shen et al. [37] proposed a smartphone-based colorimetric detection method, where images of the colored test strip were captured with the built-in camera of a smartphone and then transferred to a CIE 1931 color space for the quantitative diagnosis of pH values. But it should be noted that the CIE intensity is not in a monotonic relationship with the corresponding pH value, but a curve in the 3D color space. Moreover, a smartphone was only used in the picturing and reading process while the analysis that followed was done a computer.

Here we propose an on-chip pH regulator utilizing the diffusion properties of ionic electrolytes in solution to achieve a continuous spatial pH gradient and stable pH environment at the same time. The diffusion process is presented in detail. Based on our previous work [36], the colorimetric analysis process is carried out purely by a smartphone with no need for a computer. Simply by taking an image of the commercially available pH testing strip with built-in cameras, the colorimetric process can be completed within several seconds by the smartphone’s internal microprocessors, while the data could be sent to a network platform if users desire.

## 2. Simulation and Experimental

**Figure 1:**
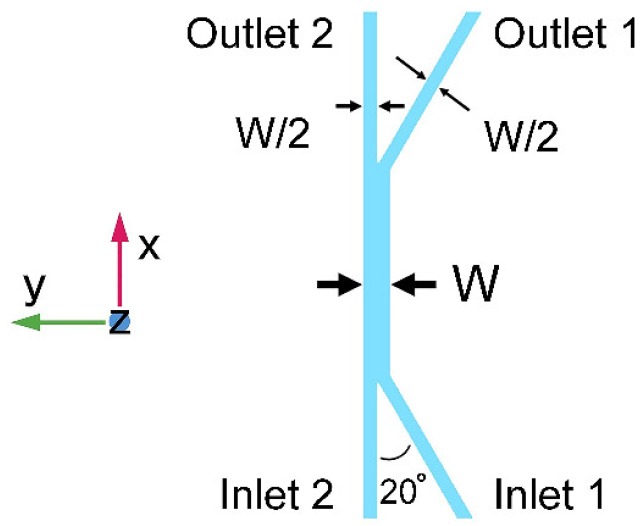
Schematic of the channel network for simulation and experiments (top view). The three-dimensional diagram of the network consists of two inlets, two outlets, and a diffusion channel where electrolyte diffuses between two laminar streams.

In this section, we propose a three-dimensional transport simulation, solved with commercially available finite element simulation software (COMSOL Multiphysics 4.3a, COMSOL Ltd., Burlington, MA, USA), for the stationary ionic concentration profile in a long straight channel. The geometric layout of the 100 μm tall (Z-dimension in Figure 1) microchannel network is schematically depicted in Figure 1, with two inlets, two outlets, and one diffusion channel. The diffusion channel is detailed with its length and width as *L* and *W*, respectively, while the width of inlet and outlet channels is W/2. The buffer stream (Stream 1, deionized water) flows into and out of the diffusion channel via Inlet 1 and Outlet 1, respectively. The solution stream (Stream 2, HCl or NaOH solution with a concentration of *n*) flows into and out of the diffusion channel via Inlet 2 and Outlet 2, respectively. Here, a larger branching angle would make the laminar features easier to become destroyed, while the wider gap between two inlets ensures a higher accuracy for the fabrication process. The value of 20° is used, as it is workable for the following investigation as well as acceptable for our fabrication process.

By introducing and maintaining proper volumetric flow rate at the terminating ends of inlet-channels, steady-state laminar flows can be generated in the diffusion channel and ionic species would diffuse through the concentration gradients. Taking the liquid as incompressible Newtonian fluid, the flow velocity field (**v**) can be described by the Navier-Stokes equation

(1) $$\rho \left(\frac{\partial v}{\partial t}+v\cdot \nabla v\right)=-\nabla p+\eta \nabla^{2}v$$

where ρ, *p* and η are the mass density, hydrostatic pressure, and dynamic viscosity of the liquid, respectively. Babaei et al. [17] proved that for micro-channels and species concentrations used in this work, the amount of species adsorbed to or desorbed from the channel wall is negligible. Hence, the modified Nernst-Planck equation for the concentration profiles considers only the presence of pumped flows and the local electric potential:

(2) $$\frac{\partial n_{i}}{\partial t}=D_{i}\nabla^{2}n_{i}+n_{i}\frac{F}{RT}D_{i}\nabla^{2}\emptyset -n_{i}\nabla v$$

here, *n* is the concentration and the subscript *i* presents H^+^, OH^−^, Na^+^, or Cl^−^ ions with diffusion coefficient *D_i_*. Poisson’s equation establishes the relationship between the local ionic concentrations and the electric potential ∅. *F*, *R*, and *T* represent the Faraday constant, molar gas constant, and temperature, respectively. The values of parameters are automatically matched according to the software, unless otherwise stated [16,38].

The experimental unit, with a channel structure as shown in Figure 1, consists of a poly(dimethylsiloxane)/glass (PDMS/glass) hybrid channel network. The PDMS microchannel network is created by a replica molding procedure, which is then bonded to a glass substrate after O_2_ plasma treatment. All micro-channels are terminated by reservoirs connecting to off-chip syringe pumps. Streams are pumped in, forming laminar flows in the diffusion channel where the electrolytes diffuse from solution stream to buffer stream, and then flow out via the two outlets. Aqueous hydrochloric acid and sodium hydroxide solutions are used as acidic and alkaline solutions, respectively, with their diffusion characteristics measured experimentally under different conditions. The outflows are collected and tested by the pH meter (PHS-3C, Shanghai INESA Scientific Instrument CO., Ltd., Shanghai, China) to quantify their ionic concentrations.

## 3. Results

### 3.1. Subsection Lateral Migration of Micro-Particles in this Laminar System

Once the laminar system is initiated, the laminar characteristics act as a virtual barrier for lateral migration of micro-particles or bio-samples in the flow with a low Reynolds number (less than 20 in this work, far from the turbulence limit in closed channels, which is approximately 2000). Considering that the most widely used bio-samples are normally in the micrometer scale and their hydrodynamic behavior in a laminar flow is dominated by their size and density, we adopted polystyrene microspheres (whose density is similar to bio-samples like cells) to verify the lateral migration of micro-particles in this laminar system.

**Figure 2:**
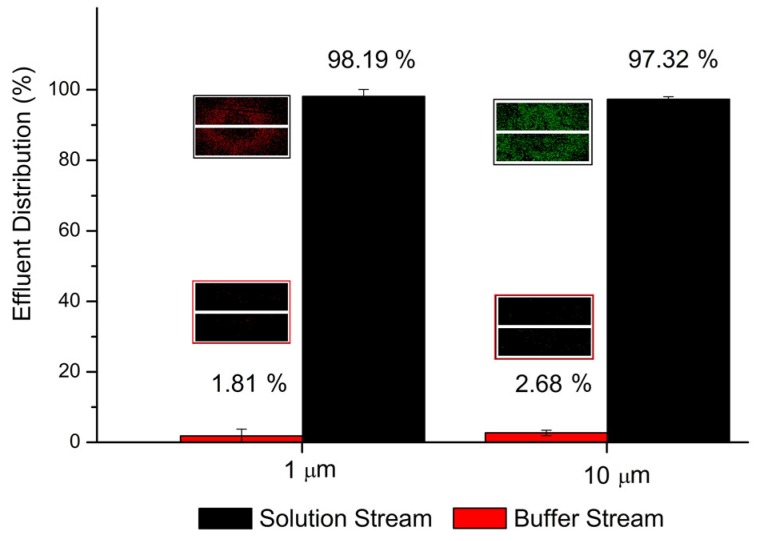
Percentage of micro-particles flowing out via two outlets. The insets are pictures of droplets from outflows under the microscope. The presented percentage is averaged from five tests.

The solution stream is intentionally mixed with polystyrene microspheres before being pumped in at a volumetric flow rate of 1 μL·min^−1^. Microspheres with a diameter of 9.9 μm (G1000, green fluorescence, Thermo Fisher Scientific, Waltham, MA, USA) and 1 μm (R0100, red fluorescence, Thermo Fisher Scientific) were adopted. Deionized water is used as buffer solution and also pumped at a volumetric flow rate of 1 μL·min^−1^. After a 10 mm long and 100 μm wide diffusion channel, outflows are collected and observed using an inverted microscope (IX 71, Olympus, Tokyo, Japan) to estimate the lateral migration of particles. As shown in Figure 2, the recovery rates for two kinds of micro beads are both higher than 97%, exhibiting the ability to operate in actual applications of analytical systems. Moreover, the flow velocity profile near the junction of the branched outlets is simulated. For a flow rate of 10 μL·min^−1^, the geometry of Y-intersection disturbs the velocity profile, but no chaotic mixing happens, due to the low Reynolds number.

### 3.2. Concentration Profiles for Hydrogen and Hydroxide Ions

**Figure 3:**
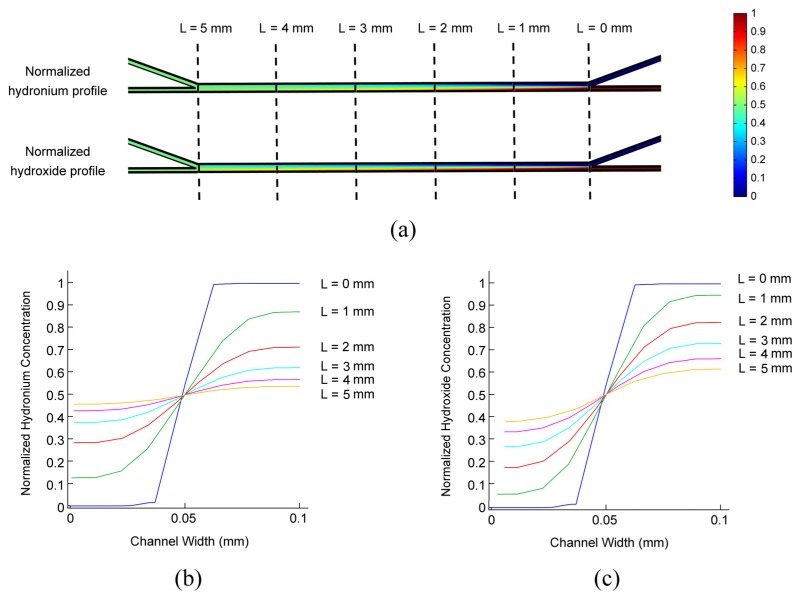
A normalized ionic concentration profile in the centric plane (Z = 50 μm in Figure 1). (**a**) Normalized ionic concentration profiles of hydronium and hydroxide. The ionic concentration is normalized by that of the flow-in solution. (**b**) The normalized hydronium concentration along the diffusion channel in the centric plane. (**c**) The normalized hydroxide concentration along the diffusion channel in the centric plane. The concentration is demonstrated in the direction of channel width (Y-direction in Figure 1). The outer channel of the water stream is noted as the start point, while the channel width is 100 μm. The volumetric flow rates for two laminar streams are 1 μL·min^−1^.

The re-distribution of ionic species occurs spontaneously and steadily within the laminar system, driven by the concentration gradient, local electric field, and superimposed pumped flows. As the spatiotemporal concentration profile is governed by Nernst-Planck, like Equation (2), we can simply normalize the concentration profiles by the initial concentrations of the two streams Thus, the ionic concentration profile drawn from one single experiment could be generalized for various conditions. The simulated salinity profiles of hydrogen and hydroxide ions in the centric plane of the microchannel network (Z = 50 μm in Figure 1) are shown in Figure 3. Two laminar streams flow through a 100 μm wide and 5 mm long diffusion channel both at a flow rate of 1 μL·min^−1^. The laminar characteristics are well maintained in the diffusion channel in the simulation, which match well with the experimental measurement. The normalized spatial distributions of hydrogen and hydroxide ions in the width direction (Y direction in Figure 1) of the diffusion channel are also presented in Figure 3b,c for L = 0, 1, 2, 3, 4, and 5 mm, respectively. As the diffusion effect dominates at low flow rates, the diffusion flux is approximately proportional to the negative of the concentration gradient. Therefore, the spatial ionic concentration is approximately in a Gaussian distribution in the channel width direction. This provides great convenience for further analysis and calculations.

### 3.3. Continuous Spatial Gradient of pH along the Diffusion Channel

**Figure 4:**
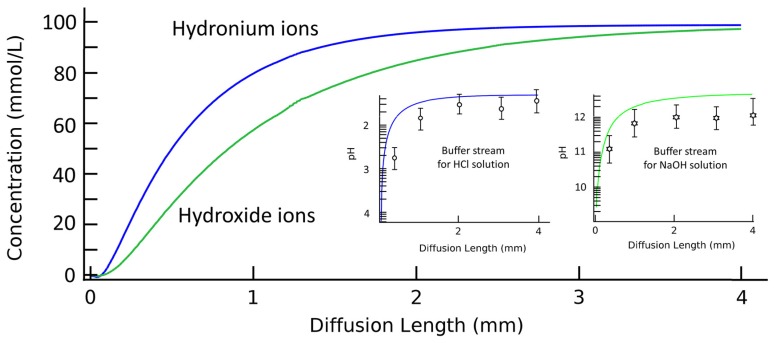
Simulation and experimental results of spatial ionic concentration and the corresponding pH gradient of the buffer stream along the diffusion channel. The simulation results are averaged from the concentration profile of the buffer stream cross-sections along the diffusion channel. Inserts are corresponding pH gradients of buffer streams along the diffusion channel when solution streams are HCl or NaOH solutions, respectively. The length of the diffusion channel is changed from 0.5 mm to 4 mm while the pH values of buffer streams are tested by a pH meter. The experimental results are demonstrated as circle and star dots in inserts. The width of diffusion channel is 100 μm. The volumetric flow rates for two laminar streams are 1 μL·min^−1^. The presented experimental results are averaged from five tests.

For a closer analysis of the spatial concentration profiles and the corresponding pH values in a bi-phase laminar system, Figure 4 illustrates experimental measurements as well as the simulation results of the spatial pH gradient along the diffusion channel. Buffer stream is pumped into a 4 mm long diffusion channel at a volumetric flow rate of 1 μL·min^−1^. Solution streams of 200 mM HCl or NaOH are pumped in for the concentration regulation of hydrogen or hydroxide, respectively. For simulation results, the ionic concentration profiles of cross-sectional views are extracted along the diffusion channel to calculate the average ionic concentrations in each cut plane. The calculated average ionic concentrations are then transferred to corresponding pH values. PDMS/glass units with different channel lengths are created to experimentally test the pH value of flowing out buffer streams. It is clear that the diffusion mechanism is effective both for pH increasing or decreasing process according to the ionic solutes in two laminar streams. The discrepancy between the experimental results from the simulation is possibly due to the dissolution of carbon dioxide, but this may require further verification. The hydrogen ions act more quickly compared to hydroxide ions for a faster ionic diffusion coefficient and migration velocity. The Gaussian distribution of ionic species in the solution thus achieves a linear function of pH values to the flow-through distance. However, temporal and spatial analysis are still needed for requested pH values. The complex ionic profiles in the channel and the device structure that needs to change with this request make it difficult to be promoted as a versatile method.

### 3.4. Regulating pH Values via Tuning Flow Rate

**Figure 5:**
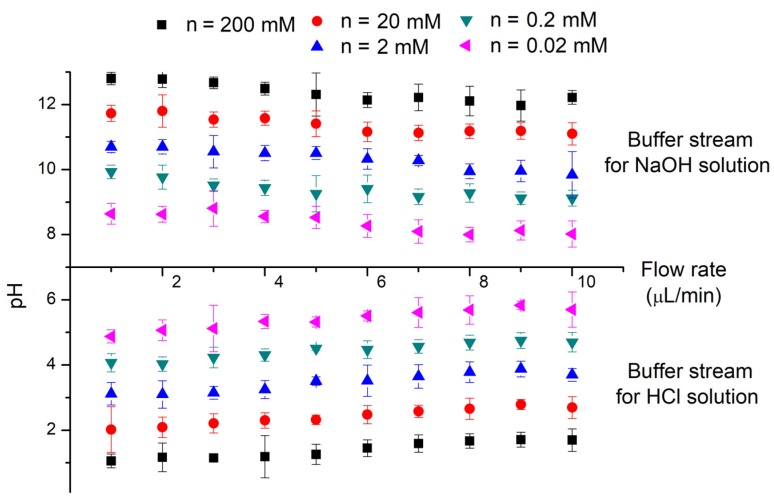
Experimentally tested pH values of flowing out buffer streams along the diffusion channel. Buffer streams (water) are pumped into a 5 mm diffusion channel with solution streams (HCl or NaOH solutions). Different concentrations of HCl and NaOH are tested under various flow rates. The width of diffusion channel is 100 μm. The volumetric flow rates for two laminar streams range from 1 μL·min^−1^ to 10 μL·min^−1^. The presented experimental results are averaged from five tests.

Considering the difficulties in regulating pH values via varying the channel length, the regulation of volumetric flow rate is more acceptable and feasible for its similar effect on the flow-through time in a fixed channel length and thus the diffusion time. Aqueous HCl and NaOH solutions with a concentration from 0.02 mM to 200 mM are introduced to the diffusion channel to decrease or increase the pH of buffer streams (deionized water), respectively. The laminar streams flow through the diffusion channel with a fixed channel length of 5 mm while the volumetric flow rate increases from 1 μL·min^−1^ to 10 μL·min^−1^. Experimental results for flowing out buffer streams are demonstrated in Figure 5, which agrees well with the numerical model. The volumetric flow rate shows comparable effects to the channel length in terms of the pH value changes. The pH value in the buffer stream show similar trends for different flow rates, though the faster flow rate calls for a longer distance to reach the same pH value compared to the slower one. However, laminar systems at a faster flow rate may need a little less diffusion time to reach the same magnitude of pH changes compared to a slower flow rate, on account of the supplement of the superimposed pumped flow, which shows its influence for flow rates larger than 10 μL·min^−1^ in our experiments. Consequently, the output pH value of outflows should be a result of both diffusion process and flow-through time through the diffusion channel. If the time needed for an even distribution is longer in comparison to the flow-through time, parameters like the flow rate and channel length that would influence the flow-through time are much more pronounced. Otherwise, the pH value of outflows should be the value corresponding to the average ionic concentration of the two initial flow-in streams.

### 3.5. Regulating pH Values for Non-Neutral Streams

**Figure 6:**
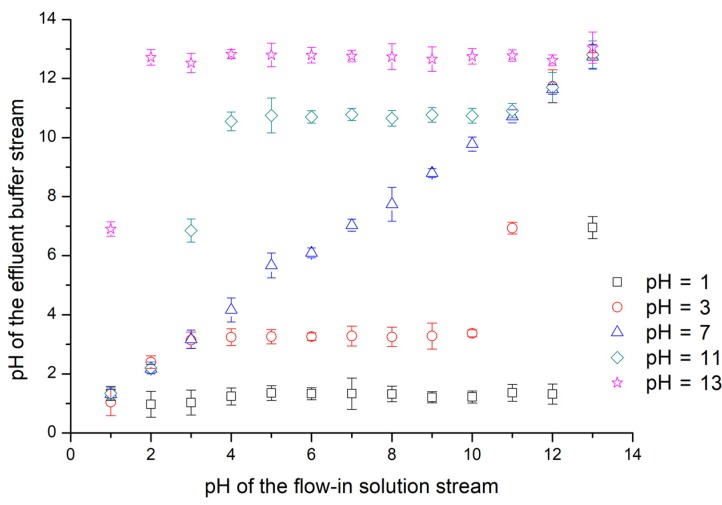
Experimental results of pH values of flowing out buffer streams along the diffusion channel. Buffer streams (HCl or NaOH solutions with a pH value of 1, 3, 7, 11 or 13) are pumped into a 10 mm diffusion channel with solution streams (HCl or NaOH solutions). The width of diffusion channel is 100 μm. The volumetric flow rates for two laminar streams are 1 μL·min^−1^. The outflows reach an even ionic distribution after the reaction between acid and alkaline in the flow-through time along the diffusion channel. The presented experimental results are averaged from five tests.

The regulating characteristics for the pH value in a bi-phase laminar system have been investigated by numerical simulation and experimental measurements under several parameters like initial concentration, channel length, and volumetric flow rate. However, results presented above are all obtained in a bi-phase system consisting of an ionic solution and a water stream, but acid or alkaline aqueous samples are more often treated in practice. Thus, liquid samples containing a certain concentration of HCl or NaOH solutes are pumped into the diffusion channel as buffer streams, whose pH values are then regulated by acid or alkaline solution streams. The final achieved pH values for flowing out buffer streams are measured and demonstrated in Figure 6, after flowing through a 10 mm long diffusion channel at a flow rate of 1 μL·min^−1^. As the corresponding concentration of hydrogen and hydroxide ions in the solution has to change tenfold to increase or decrease its pH value by 1 unit, the final achieved hydrogen or hydroxide concentration should be consistent with the solution with the dominating initial ionic concentration, even if the interfacial reaction of hydrogen and hydroxide ions is taken into account. The long channel and low flow rate provide enough time for the diffusion process to reach an even distribution, making it possible to estimate the pH value of the outflow with only the ionic concentrations of the two flow-in streams. Considering the difficulties of modulating pH values via channel length or volumetric flow rate, this approach is much easier and more convenient with no need to calculate or measure the complicated ionic profile along the diffusion channel.

### 3.6. Smartphone-Based Colorimetric pH Detection

Previously developed paper-based microfluidic devices or test strips are normally viewed by the naked eye, which makes it difficult to achieve precise measurement results. Several works have proposed the use of a smartphone to replace bulky detection instruments. However, the smartphones were only used in picturing and reading processes, while the ensuing analyses still proceeded on computers. In this work, we further propose a smartphone-based colorimetric method for water samples with the help of commercially available pH test strips as a simple quantitative point-of-care colorimetric analysis. One droplet is extracted from the outflow to a test strip for the colorimetric detection. A picture of the colored region is captured with the camera of a Sony Xperia Z3 Compact smartphone and later processed through a developed Android application for quantification of the corresponding pH value. Briefly, images of the test strip were captured by the camera on the smartphone. A circular area with a radius of one millimeter is taken at the center of the colored area in the photo images. The red, green, and blue values of each pixel in this area were extracted from the smartphone and added up separately. The RGB values could be processed then in the microprocessor of the smartphone for future usage. The only used light source is the built-in flash of the smartphone camera and the detection interference could be further compensated with a self-calibration technique using a standard solution with known pH values, as we demonstrated before [36]. 

**Figure 7:**
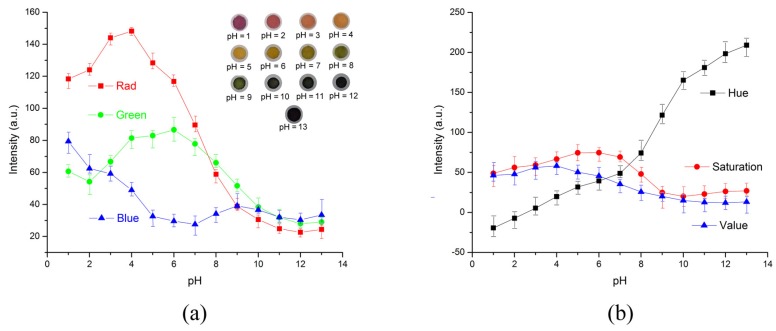
Intensities of pH test strips at each pH value in different color spaces. (**a**) RGB intensities of pH test strips at each pH value. None of the individual channel intensities correlate with the pH values in a monotonic function. Inserts are photos of testing strips taken by the smartphone. (**b**) Colors of pH test strips in HSV color space. The intensity of the H coordinate appears to be a monotonic function of the test strips’ chromaticity values. The presented experimental results are averaged from five tests.

**Figure 8:**
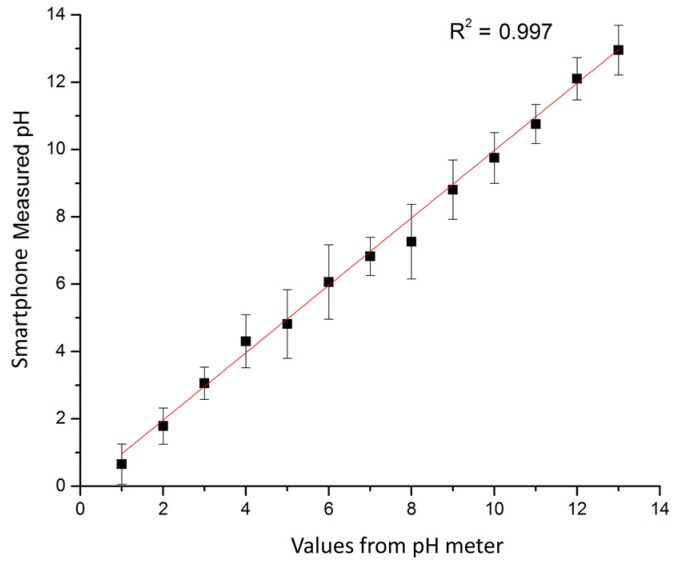
Standard curve of the smartphone processor response to different pH solutions compared to a commercial pH meter. The presented experimental results are averaged from five tests.

The RGB values of the test strips are plotted in Figure 7a for calibration solutions with pH values increasing from 1 to 12. The pictures of colored test strips are inserted to illustrate their colorimetric results by naked eyes. Though our previous work could ensure the accuracy [36], RGB intensities for pH increasing from 1 to 12 exhibit little discernible trends and are difficult to correlate with corresponding pH values. To overcome the non-monotonic relationship and inadequacies of the direct RGB method, the H (hue) coordinate of the HSV color space transferred from conventional RGB values is adopted as shown in Figure 7b. The intensity of the H coordinate appears to be a monotonic function of the test strips’ chromaticity values, which intuitively reflects the color change of the test strip and the corresponding pH values. Thus, the H coordinate is chosen as the detected parameter for this colorimetric analyzing process. Figure 8 illustrates the standard curve for measuring pH in a range from 1 to 12, demonstrating that this smartphone-based colorimetric detection method is versatile and works well for pH measurement. The observed sensitivity could be improved by using narrow range and high precise pH test strips for more accurate measurements. Besides, it should be noted that the calibration curves and testing accuracy are also dependent on the smartphone complementary metal-oxide-semiconductor (CMOS) imaging chip. Relative results about the difference between several brands or types of smartphones are proposed previously in our works [36]. Consequently, this smartphone-based colorimetric mechanism could be a simple way for the real-time monitoring of the output pH value. The detection result is used reversely as a feedback to adjust relative parameters, like flow rates, to achieve the desired pH value.

## 4. Conclusions

We have demonstrated a simple and adaptive microfluidic system that enables the on-demand regulation of pH values in laminar streams utilizing a diffusion mechanism. The continuous spatial gradients of hydrogen and hydroxide ions are achieved in a micro-channel network, while an outflow of specific and stable pH values is simultaneously achieved. The final achieved pH values and gradients could be well controlled by the channel length, volumetric flow rate, and initial ionic concentration of flow-in streams. A numerical model is proposed for the quantitative description of the ionic profile and thus the pH values, which is in good agreement with experimental measurements. To make it a better portable device, a smartphone-based colorimetric analyzing method is proposed. Our diffusion-based pH regulator is stable, easy to make, and flexible in operation, with no contamination from the direct contact. Applications could be found in the fields of biological assay, protein crystallization, enzyme assay, and so on.
